# Ferroptosis and futile recanalization after mechanical thrombectomy in acute ischemic stroke: mechanisms, risk factors, predictive models and therapeutic interventions

**DOI:** 10.3389/ebm.2026.10997

**Published:** 2026-05-01

**Authors:** Yuexin Wu, Cuiying Zhang, Yangyang Peng, Longlong Liu

**Affiliations:** Emergency Department, Binzhou People’s Hospital, Binzhou, Shandong, China

**Keywords:** acute ischemic stroke, ferroptosis, futile recanalization, iron metabolism, mechanical thrombectomy

## Abstract

Acute ischemic stroke (AIS) remains a leading cause of mortality and long-term disability worldwide. Endovascular mechanical thrombectomy (EVT) has revolutionized stroke treatment by enabling rapid recanalization of occluded cerebral vessels. However, a significant proportion of patients experience futile recanalization, where successful vessel reopening fails to translate into favorable functional outcomes. Emerging evidence highlights ferroptosis, a regulated form of cell death driven by iron-dependent lipid peroxidation, as a critical mechanism contributing to neuronal damage in ischemic stroke, particularly during reperfusion injury. This comprehensive review examines the intricate relationship between ferroptosis and futile recanalization following mechanical thrombectomy. We systematically explore the molecular mechanisms underlying ferroptosis in the context of cerebral ischemia-reperfusion injury, including iron metabolism dysregulation, lipid peroxidation cascades, glutathione depletion, and mitochondrial dysfunction. Multiple factors contribute to futile recanalization, including patient demographics (age, comorbidities), stroke characteristics (severity, infarct volume, collateral status), procedural variables (time to treatment, recanalization quality), and post-procedural complications (hemorrhagic transformation, reperfusion injury). We review current predictive models, including nomograms and machine learning algorithms, that integrate clinical, radiological, and biomarker data to stratify patient risk. Importantly, we discuss potential therapeutic interventions targeting ferroptosis pathways, such as iron chelators, lipophilic antioxidants, and ferroptosis-specific inhibitors. The integration of ferroptosis biomarkers into predictive models may enhance risk stratification and guide personalized treatment strategies. Future research should focus on validating ferroptosis-targeted therapies in clinical trials, developing real-time monitoring techniques, and establishing standardized protocols for neuroprotective interventions during mechanical thrombectomy. A deeper understanding of ferroptosis mechanisms and their contribution to futile recanalization may pave the way for novel therapeutic approaches to improve stroke outcomes.

## Impact statement

We are pleased to submit our review manuscript entitled “Ferroptosis and Futile Recanalization after Mechanical Thrombectomy in Acute Ischemic Stroke: Mechanisms, Risk Factors, Predictive Models and Therapeutic Interventions” for your consideration. This comprehensive review explores the critical role of ferroptosis—a regulated, iron-dependent cell death pathway—in contributing to poor functional outcomes (futile recanalization) following successful endovascular thrombectomy. We systematically synthesize current evidence on molecular mechanisms, clinical predictors, emerging prognostic models, and promising therapeutic strategies targeting ferroptosis. By integrating preclinical insights with clinical observations, this work aims to advance the understanding of reperfusion injury and highlight novel neuroprotective approaches to improve stroke outcomes. We believe this review will be of significant interest to your readers and contribute to ongoing translational research in stroke neurology.

## Introduction

Acute ischemic stroke (AIS) represents one of the most devastating neurological emergencies, ranking as the second leading cause of death and the third leading cause of disability-adjusted life years worldwide [1–3]. The abrupt interruption of cerebral blood flow triggers a complex pathophysiological cascade, including excitotoxicity, oxidative stress, inflammation, and cell death, ultimately resulting in neuronal injury and functional impairment [4]. Despite significant advances in acute stroke management, including intravenous thrombolysis and endovascular mechanical thrombectomy (EVT), a substantial proportion of patients continue to experience poor functional outcomes [5].

The introduction of EVT has revolutionized the treatment paradigm for patients with large vessel occlusion (LVO) stroke. Multiple landmark randomized controlled trials, including MR CLEAN, EXTEND-IA, ESCAPE, SWIFT PRIME, and REVASCAT, have demonstrated the superiority of EVT over medical therapy alone, with significant improvements in functional independence and reduced mortality [6, 7]. Subsequent trials such as DAWN and DEFUSE-3 further expanded the therapeutic window, showing benefits in carefully selected patients up to 24 h from symptom onset [8, 9]. Despite these remarkable advances in recanalization rates, achieving successful angiographic recanalization (modified Treatment in Cerebral Ischemia [mTICI] score 2b-3) does not guarantee favorable functional outcomes [10, 11].

This phenomenon, termed “futile recanalization,” occurs when successful vessel recanalization fails to translate into meaningful clinical improvement, typically defined as a modified Rankin Scale (mRS) score of 3–6 at 90 days despite achieving mTICI 2b-3 reperfusion [12, 13]. The incidence of futile recanalization ranges from 30% to 68% across different studies, representing a significant clinical challenge and highlighting the complexity of ischemic stroke pathophysiology beyond vessel occlusion [14, 15]. Understanding the mechanisms underlying futile recanalization is crucial for developing strategies to optimize treatment outcomes and identify patients who are most likely to benefit from aggressive revascularization therapy.

Recent advances in cell death research have unveiled ferroptosis as a novel and distinct form of regulated cell death that plays a critical role in ischemic stroke pathophysiology [16–18]. Unlike apoptosis, necrosis, or autophagy, ferroptosis is characterized by iron-dependent accumulation of lipid peroxides, resulting from the failure of cellular antioxidant defenses [19]. This form of cell death was first described by Dixon et al. in 2012 and has since emerged as a significant contributor to neuronal injury in various neurological disorders, including stroke, traumatic brain injury, and neurodegenerative diseases [7].

The relevance of ferroptosis to stroke becomes particularly evident in the context of ischemia-reperfusion injury. While prompt restoration of blood flow through mechanical thrombectomy is essential to salvage ischemic tissue, reperfusion paradoxically triggers additional injury mechanisms, including oxidative stress, calcium overload, mitochondrial dysfunction, and inflammatory responses [20]. Ferroptosis appears to be a key mediator of reperfusion injury, as the restoration of blood flow delivers oxygen and iron to ischemic tissue, creating conditions conducive to lipid peroxidation and ferroptotic cell death [21]. This dual-edged nature of reperfusion may partially explain the phenomenon of futile recanalization, where successful vessel reopening fails to prevent ongoing neuronal damage.

Multiple lines of evidence support the involvement of ferroptosis in acute ischemic stroke. Experimental studies have demonstrated increased markers of ferroptosis, including elevated iron levels, lipid peroxidation products (such as malondialdehyde and 4-hydroxynonenal), and depleted glutathione, in ischemic brain tissue [22]. Furthermore, pharmacological inhibition of ferroptosis using specific inhibitors like ferrostatin-1 and liproxstatin-1 has shown neuroprotective effects in preclinical stroke models, reducing infarct volume and improving functional outcomes [22]. Genetic manipulations targeting key ferroptosis regulators, such as glutathione peroxidase 4 (GPX4) and ferroptosis suppressor protein 1 (FSP1), have similarly demonstrated the causal role of ferroptosis in stroke pathophysiology [23].

The molecular mechanisms governing ferroptosis involve a complex interplay of iron metabolism, lipid peroxidation, and antioxidant defense systems. Iron homeostasis is tightly regulated under physiological conditions through coordinated expression of iron import proteins (transferrin receptor 1), storage proteins (ferritin), and export proteins (ferroportin) [24]. However, cerebral ischemia and subsequent reperfusion disrupt this delicate balance, leading to increased intracellular labile iron pools that catalyze the Fenton reaction, generating highly reactive hydroxyl radicals [20]. These radicals initiate lipid peroxidation of polyunsaturated fatty acids (PUFAs) in cellular membranes, particularly phosphatidylethanolamines containing arachidonic acid or adrenic acid [25, 26]. The accumulation of lipid peroxides ultimately leads to membrane damage and ferroptotic cell death.

Cellular antioxidant defense mechanisms, particularly the GPX4-glutathione axis and the FSP1-CoQ10 system, serve as critical barriers against ferroptosis [27]. GPX4, a selenoprotein, reduces lipid hydroperoxides to lipid alcohols using glutathione (GSH) as a cofactor, thereby preventing the propagation of lipid peroxidation [28]. System xc^-^, a cystine/glutamate antiporter composed of SLC7A11 and SLC3A2 subunits, imports cystine for GSH synthesis, representing another key component of the anti-ferroptotic defense [29, 30]. During ischemic stroke, depletion of GSH, inactivation of GPX4, or dysfunction of system xc^-^ sensitizes neurons to ferroptotic cell death [31, 32]. Understanding these molecular mechanisms provides a foundation for developing targeted therapeutic interventions to prevent ferroptosis-mediated injury in stroke patients undergoing mechanical thrombectomy.

This comprehensive review aims to elucidate the role of ferroptosis in futile recanalization following mechanical thrombectomy for acute ischemic stroke. We systematically examine the molecular mechanisms of ferroptosis in the context of cerebral ischemia-reperfusion injury, explore clinical and radiological factors associated with futile recanalization, review current predictive models, and discuss potential therapeutic interventions targeting ferroptosis pathways. By integrating basic science discoveries with clinical observations, we hope to provide a framework for future research and therapeutic development aimed at optimizing outcomes after mechanical thrombectomy through ferroptosis-targeted neuroprotection.

## Molecular mechanisms of ferroptosis in cerebral ischemia

### Iron metabolism and dysregulation in ischemic stroke

Iron is an essential cofactor for numerous biological processes, including oxygen transport, DNA synthesis, and mitochondrial respiration. However, its redox-active nature makes iron a double-edged sword, as excessive free iron can catalyze the formation of reactive oxygen species (ROS) through Fenton chemistry [30]. In the brain, iron homeostasis is particularly critical, as neurons are highly vulnerable to oxidative damage due to their high metabolic rate, abundant polyunsaturated fatty acids, and relatively limited antioxidant capacity [33].

Under physiological conditions, iron levels in the brain are tightly regulated through the coordinated action of multiple proteins. Transferrin receptor 1 (TfR1) mediates cellular iron uptake by binding iron-loaded transferrin, which is subsequently internalized via clathrin-mediated endocytosis [34]. Once inside the cell, iron is released from transferrin in the acidic endosomal environment and reduced from Fe^3+^ to Fe^2+^ by the ferrireductase STEAP3. The Fe^2+^ is then transported into the cytosol by divalent metal transporter 1 (DMT1), where it enters the labile iron pool (LIP) - a redox-active pool of chelatable iron that serves as the source for iron incorporation into various cellular processes [29].

Excess intracellular iron is stored in ferritin, a multi-subunit protein complex composed of heavy (FTH1) and light (FTL) chains that can sequester up to 4,500 iron atoms in a non-toxic ferric form [28]. Ferroportin (FPN), the sole known mammalian iron exporter, mediates cellular iron efflux and is regulated by hepcidin, a systemic iron regulatory hormone [33]. This intricate regulatory network maintains iron homeostasis and prevents iron-mediated toxicity under normal conditions.

Cerebral ischemia profoundly disrupts iron homeostasis through multiple mechanisms. During ischemia, the breakdown of the blood-brain barrier allows extravasation of iron-containing proteins, including hemoglobin and transferrin, into the brain parenchyma [34]. Simultaneously, hemorrhagic transformation, a common complication following ischemic stroke, particularly after reperfusion therapy, introduces large amounts of hemoglobin-derived iron into brain tissue [29]. Red blood cell lysis releases hemoglobin, which is subsequently broken down by heme oxygenase-1 (HO-1), liberating free iron [30]. Interestingly, the role of heme oxygenase-1 (HO-1) in ischemic stroke appears to be dual and highly context-dependent. Under physiological or mildly stressed conditions, HO-1 is generally regarded as cytoprotective, largely due to its antioxidant, anti-inflammatory, and heme-detoxifying effects. However, during acute ischemia–reperfusion (I/R) injury, excessive or dysregulated HO-1 induction may paradoxically facilitate ferroptosis by increasing intracellular iron availability as a consequence of heme degradation. The resulting surge in free ferrous iron expands the labile iron pool and accelerates Fenton chemistry, thereby amplifying lipid peroxidation and aggravating neuronal injury [31]. Thus, while moderate HO-1 activity may confer endogenous protection, uncontrolled activation in the reperfusion phase has the potential to exacerbate ferroptosis-mediated neuronal damage, which may in turn contribute to futile recanalization despite successful vessel reopening [32].

Reperfusion following mechanical thrombectomy creates conditions particularly conducive to ferroptosis. The sudden restoration of oxygen supply in the presence of elevated iron levels creates an ideal environment for Fenton chemistry: Fe^2+^ + H_2_O_2_ → Fe^3+^ + OH^−^ + •OH. The hydroxyl radical (•OH) generated is among the most reactive oxygen species, capable of initiating lipid peroxidation cascades that culminate in ferroptotic cell death. Moreover, ischemia-reperfusion injury triggers inflammatory responses that further exacerbate iron dysregulation. Activated microglia and infiltrating macrophages release pro-inflammatory cytokines that upregulate TfR1 expression and downregulate ferroportin, promoting cellular iron accumulation [29].

**FIGURE 1:**
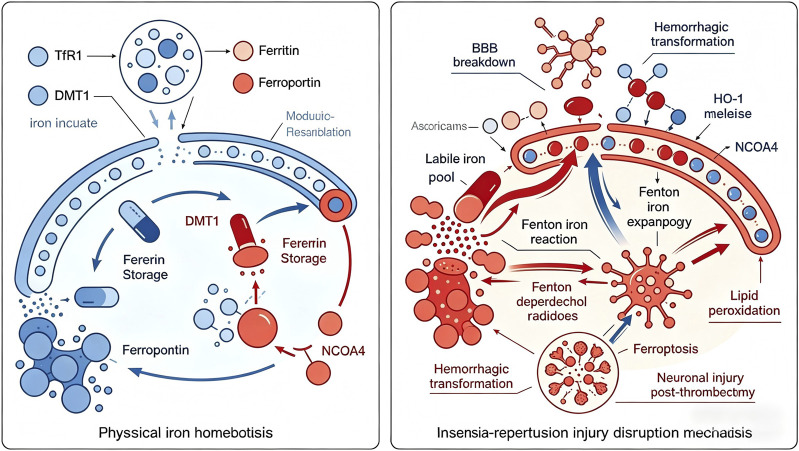
Iron Metabolism Dysregulation and Ferroptosis in Cerebral Ischemia-Reperfusion Injury. Left panel: Under physiological conditions, neuronal iron homeostasis is maintained through coordinated TfR1-mediated uptake, DMT1 transport, ferritin storage, and ferroportin export. Right panel: Ischemia-reperfusion injury disrupts iron homeostasis via multiple mechanisms including blood-brain barrier breakdown, hemorrhagic transformation, HO-1-mediated iron release, and NCOA4-driven ferritinophagy, leading to labile iron pool expansion. Excess free iron (Fe^2+^) reacts with reperfusion oxygen via Fenton chemistry, generating hydroxyl radicals that initiate lipid peroxidation and trigger ferroptosis, contributing to neuronal injury and futile recanalization following mechanical thrombectomy.

Recent studies have identified ferritinophagy, the selective autophagic degradation of ferritin, as a key mechanism driving ferroptosis in ischemic stroke [30]. Nuclear receptor coactivator 4 (NCOA4) acts as a cargo receptor for ferritinophagy, binding to ferritin heavy chain and delivering it to lysosomes for degradation [35]. While ferritinophagy serves an important physiological role in mobilizing iron stores under iron-deficient conditions, excessive ferritinophagy during ischemia-reperfusion injury liberates large amounts of iron from ferritin, expanding the labile iron pool and promoting ferroptosis. Genetic or pharmacological inhibition of NCOA4 has been shown to attenuate ferroptosis and reduce ischemic brain injury in experimental models (Figure 1) [36].

### Lipid peroxidation and membrane damage

Lipid peroxidation represents the biochemical hallmark of ferroptosis and is initiated by the oxidation of polyunsaturated fatty acids (PUFAs) in cellular membranes. Unlike other forms of cell death that may involve lipid peroxidation as a secondary event, ferroptosis is defined by the overwhelming accumulation of lipid peroxides to levels that exceed cellular repair capacity. The process is highly specific, targeting phospholipids containing PUFA tails, particularly those esterified to arachidonic acid (AA, 20:4) or adrenic acid (AdA, 22:4) [37].

The lipid peroxidation cascade begins with the abstraction of a bis-allylic hydrogen atom from PUFAs by reactive oxygen species, particularly hydroxyl radicals generated through Fenton chemistry. This produces a carbon-centered lipid radical (L•), which rapidly reacts with molecular oxygen to form a lipid peroxyl radical (LOO•). The lipid peroxyl radical can then abstract a hydrogen atom from an adjacent PUFA, generating a lipid hydroperoxide (LOOH) and propagating a chain reaction that amplifies oxidative damage [37]. This autocatalytic nature of lipid peroxidation explains why even small amounts of initial oxidative stress can trigger extensive membrane damage.

Recent lipidomics studies have revealed that ferroptosis selectively targets specific phospholipid species. Acyl-CoA synthetase long-chain family member 4 (ACSL4) plays a crucial role in determining ferroptosis sensitivity by preferentially incorporating AA and AdA into phospholipids. Lysophosphatidylcholine acyltransferase 3 (LPCAT3) further esterifies these PUFAs specifically into phosphatidylethanolamine (PE), generating PE-AA and PE-AdA species that are highly susceptible to peroxidation [38]. Cells lacking ACSL4 or LPCAT3 show remarkable resistance to ferroptosis, even in the presence of canonical ferroptosis inducers, highlighting the critical importance of PUFA-containing phospholipids in the ferroptotic process [39].

In the context of ischemic stroke and reperfusion, multiple factors converge to promote lipid peroxidation. First, ischemia depletes cellular ATP, impairing energy-dependent antioxidant systems and phospholipid repair mechanisms [35]. Second, reperfusion generates a burst of reactive oxygen species through multiple sources, including mitochondrial electron transport chain dysfunction, NADPH oxidase activation, and xanthine oxidase-mediated superoxide production [36]. Third, the inflammatory response following stroke upregulates lipoxygenases (LOXs), particularly 15-LOX, which directly catalyze the peroxidation of PUFA-containing phospholipids [38]. 15-LOX has been specifically implicated in ferroptotic cell death, and its inhibition provides neuroprotection in experimental stroke models [39].

**FIGURE 2:**
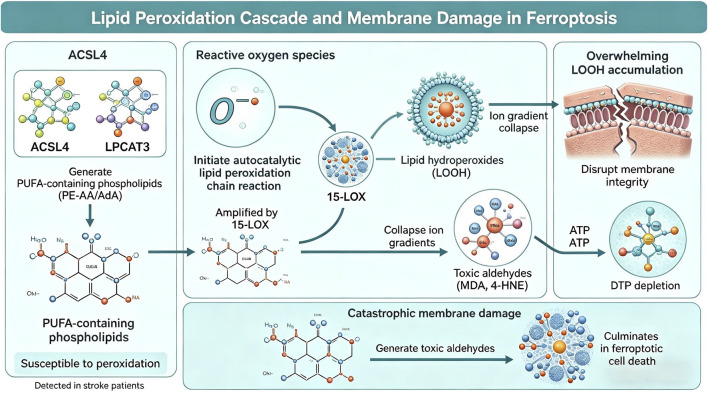
Lipid Peroxidation Cascade and Membrane Damage in Ferroptosis. ACSL4 and LPCAT3 generate PUFA-containing phospholipids (PE-AA/AdA) susceptible to peroxidation. Reactive oxygen species initiate an autocatalytic lipid peroxidation chain reaction, amplified by 15-LOX, producing lipid hydroperoxides (LOOH). Overwhelming LOOH accumulation disrupts membrane integrity, collapses ion gradients, generates toxic aldehydes (MDA, 4-HNE), and depletes ATP, culminating in catastrophic membrane damage and ferroptotic cell death detected in stroke patients.

The accumulation of lipid hydroperoxides causes catastrophic membrane damage through several mechanisms. Lipid peroxidation disrupts membrane fluidity and integrity, leading to loss of ion gradients, impaired function of membrane-bound proteins, and ultimately cell lysis [38]. Additionally, lipid peroxidation products, such as malondialdehyde (MDA) and 4-hydroxynonenal (4-HNE), are highly reactive aldehydes that can modify proteins and DNA, amplifying cellular dysfunction [40]. These lipid-derived aldehydes serve as biomarkers of oxidative stress and ferroptosis, with elevated levels detected in both experimental stroke models and human stroke patients (Figure 2).

### Glutathione peroxidase 4 and antioxidant defense systems

Glutathione peroxidase 4 (GPX4) represents the central guardian against ferroptosis, functioning as the only enzyme capable of directly reducing lipid hydroperoxides within biological membranes [39]. Unlike other members of the glutathione peroxidase family that primarily reduce hydrogen peroxide and other small molecular oxidants, GPX4’s unique ability to access and reduce phospholipid hydroperoxides makes it indispensable for preventing ferroptosis [35]. The critical importance of GPX4 is underscored by the fact that complete genetic deletion of GPX4 is embryonically lethal in mice, while conditional deletion in specific tissues leads to spontaneous ferroptosis [36].

GPX4 functions through a catalytic mechanism that requires reduced glutathione (GSH) as a cofactor and selenium in the form of selenocysteine at its active site [37]. The enzyme reduces lipid hydroperoxides (LOOH) to their corresponding alcohols (LOH), while oxidizing GSH to glutathione disulfide (GSSG). The GSSG is then reduced back to GSH by glutathione reductase using NADPH, completing the antioxidant cycle [38]. Therefore, the efficacy of GPX4 in preventing ferroptosis depends not only on adequate GPX4 expression and activity but also on sufficient availability of GSH, the proper functioning of glutathione reductase, and adequate NADPH supply [39].

During cerebral ischemia and reperfusion, multiple factors conspire to impair GPX4 function and cellular antioxidant capacity. First, ischemia-induced energy depletion reduces NADPH levels, limiting the regeneration of GSH from GSSG. Second, oxidative stress during reperfusion can directly oxidize and inactivate GPX4, rendering it unable to reduce lipid peroxides [41]. Third, selenium deficiency, whether due to dietary insufficiency or increased utilization during oxidative stress, can compromise GPX4 activity, as selenocysteine incorporation is essential for its catalytic function [42].

The synthesis of glutathione, a tripeptide composed of glutamate, cysteine, and glycine, is rate-limited by cysteine availability. System xc^-^, a sodium-independent cystine/glutamate antiporter located on the plasma membrane, plays a crucial role in providing cysteine for GSH synthesis. This transporter imports cystine (the oxidized form of cysteine) from the extracellular space in exchange for intracellular glutamate. Once inside the cell, cystine is rapidly reduced to cysteine, which is then incorporated into GSH by the sequential action of glutamate-cysteine ligase (GCL) and glutathione synthetase (GS) [43].

System xc^-^ comprises two subunits: SLC7A11 (also known as xCT), the light chain responsible for substrate transport, and SLC3A2 (also known as 4F2hc or CD98), a heavy chain chaperone protein required for proper trafficking and stabilization of SLC7A11 at the plasma membrane [44]. Inhibition of system xc^-^, whether through genetic deletion, pharmacological blockade (e.g., by erastin or sulfasalazine), or downregulation in pathological conditions, leads to cysteine depletion, GSH exhaustion, and ultimately ferroptosis [45].

In ischemic stroke, system xc^-^ activity may be compromised through multiple mechanisms. Glutamate excitotoxicity, a well-established consequence of cerebral ischemia, results in massive accumulation of extracellular glutamate, which can inhibit system xc^-^ by reducing the driving force for cystine import. Additionally, inflammatory mediators released during ischemia-reperfusion injury can regulate SLC7A11 expression. For instance, interferon-gamma (IFN-γ) has been shown to downregulate SLC7A11 through JAK-STAT1 signaling, potentially sensitizing cells to ferroptosis [46]. Conversely, activation of the Nrf2 (nuclear factor erythroid 2-related factor 2) pathway, a master regulator of antioxidant responses, can upregulate SLC7A11 expression, enhancing cellular resistance to ferroptosis [47].

Beyond the GPX4-GSH axis, recent research has identified additional anti-ferroptotic pathways that provide redundancy in cellular defense mechanisms. The FSP1 (ferroptosis suppressor protein 1)-CoQ10 (coenzyme Q10) system represents a parallel pathway that operates independently of GSH [48]. FSP1, also known as AIFM2, functions as an oxidoreductase that reduces CoQ10 to its active antioxidant form, ubiquinol, using NADPH as a cofactor. Ubiquinol can then directly reduce lipid peroxyl radicals, thereby inhibiting the propagation of lipid peroxidation [49]. Importantly, FSP1 can compensate for GPX4 deficiency, and cells expressing high levels of FSP1 show resistance to GPX4 inhibition [50].

**FIGURE 3:**
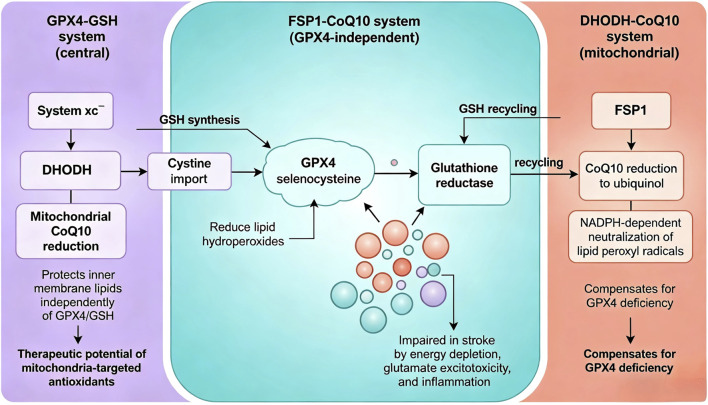
Three Complementary Antioxidant Pathways Against Ferroptosis. GPX4-GSH system (central): System xc^-^ imports cystine for GSH synthesis, which GPX4 uses with selenocysteine to reduce lipid hydroperoxides, recycled by glutathione reductase; impaired in stroke by energy depletion, glutamate excitotoxicity, and inflammation. FSP1-CoQ10 system (GPX4-independent): FSP1 reduces CoQ10 to ubiquinol using NADPH, directly neutralizing lipid peroxyl radicals and compensating for GPX4 deficiency. DHODH-CoQ10 system (mitochondrial): DHODH reduces mitochondrial CoQ10, protecting inner membrane lipids independently of GPX4/GSH, highlighting therapeutic potential of mitochondria-targeted antioxidants.

More recently, the DHODH (dihydroorotate dehydrogenase)-CoQ10 system has been identified as another GPX4-independent ferroptosis suppression mechanism [51]. DHODH, a mitochondrial enzyme involved in pyrimidine biosynthesis, can reduce CoQ10 within the mitochondrial inner membrane, protecting mitochondrial lipids from peroxidation [46]. This discovery highlights the importance of mitochondrial integrity in ferroptosis regulation and suggests that mitochondria-targeted antioxidants may offer therapeutic benefits in preventing ferroptosis-mediated injury during ischemic stroke (Figure 3).

## Ferroptosis in cerebral ischemia-reperfusion injury and futile recanalization

### Temporal dynamics of ferroptosis following reperfusion

The temporal profile of ferroptosis following cerebral ischemia-reperfusion injury reveals a complex biphasic pattern that begins during ischemia but dramatically accelerates upon reperfusion [47]. During the acute ischemic phase, energy depletion and glutamate excitotoxicity initiate early cellular stress, but the full manifestation of ferroptosis requires the oxygen and iron availability that comes with reperfusion [48]. This paradoxical nature of reperfusion injury, where restoration of blood flow exacerbates tissue damage, has long been recognized, but the specific role of ferroptosis in this process has only recently been elucidated [49].

Experimental studies using middle cerebral artery occlusion (MCAO) models in rodents have demonstrated that markers of ferroptosis, including elevated lipid peroxidation products and depleted GSH, begin to rise within hours of reperfusion [51]. The expression of ferroptosis-related genes shows dynamic changes, with downregulation of GPX4 and SLC7A11 and upregulation of ACSL4 and transferrin receptor observed in the peri-infarct region during the first 24–72 h post-reperfusion. Importantly, the timing of ferroptosis coincides with the critical period when clinical decisions regarding mechanical thrombectomy are made, suggesting that peri-procedural interventions targeting ferroptosis may be particularly effective [47].

The spatial distribution of ferroptosis in post-ischemic brain tissue provides additional insights into its role in futile recanalization. While the infarct core undergoes rapid necrotic death during ischemia, the penumbra—the potentially salvageable tissue surrounding the core—appears particularly vulnerable to ferroptotic cell death following reperfusion [52]. This observation is crucial for understanding futile recanalization, as successful vessel reopening aims to salvage the penumbra, but ferroptosis-mediated injury in this region may prevent functional recovery despite restored blood flow [49].

Furthermore, the delayed nature of ferroptosis creates a therapeutic window that extends beyond the hyperacute phase of stroke treatment. While mechanical thrombectomy primarily addresses the acute vessel occlusion, ferroptosis continues to contribute to neuronal damage for days after reperfusion, potentially explaining why some patients deteriorate despite successful early recanalization [51]. This extended time course suggests that anti-ferroptotic therapies could be administered not only during the thrombectomy procedure but also in the post-procedural period, offering multiple opportunities for intervention [46].

The temporal relationship between ferroptosis activation and therapeutic intervention represents a critical consideration for clinical translation. Based on experimental evidence from MCAO models, ferroptosis markers begin rising within 2–4 h post-reperfusion and peak at 24–72 h, suggesting multiple intervention opportunities [53]. Three potential timing strategies emerge: (1) prophylactic administration immediately before or during thrombectomy to preemptively block ferroptosis pathways; (2) concurrent administration during the procedure to target the acute reperfusion phase; and (3) post-procedural administration within 24 h to address the delayed ferroptotic injury phase [54]. The extended time course of ferroptosis, unlike the rapid necrotic core formation, provides a clinically feasible window that extends beyond the hyperacute thrombectomy period, making this approach practically implementable in real-world settings.

### Ferroptosis as a mechanistic link to futile recanalization

The phenomenon of futile recanalization, where successful angiographic vessel reopening fails to translate into favorable functional outcomes, represents one of the most frustrating challenges in acute stroke management [47]. While multiple factors contribute to futile recanalization—including advanced age, high baseline NIHSS, large infarct core, poor collaterals, and hemorrhagic transformation—the underlying cellular and molecular mechanisms remain incompletely understood. Emerging evidence suggests that ferroptosis may serve as a critical mechanistic link connecting successful recanalization to poor outcomes [52].

Several lines of evidence support the role of ferroptosis in futile recanalization. First, biomarker studies in stroke patients have shown that elevated levels of ferroptosis markers, including serum iron, ferritin, lipid peroxidation products (MDA, 4-HNE), and reduced GSH/GSSG ratio, correlate with poor functional outcomes despite successful reperfusion [55]. Patients with higher baseline serum ferritin levels, reflecting increased iron stores, show significantly higher rates of futile recanalization after mechanical thrombectomy, independent of other risk factors. Similarly, genetic polymorphisms affecting iron metabolism and antioxidant capacity have been associated with variable responses to reperfusion therapy [56].

Second, advanced neuroimaging studies have revealed patterns consistent with ferroptosis-mediated injury in patients with futile recanalization. Susceptibility-weighted imaging (SWI) and quantitative susceptibility mapping (QSM), which are sensitive to iron deposition, show increased signal in the peri-infarct region of patients with poor outcomes after thrombectomy [57]. These imaging findings correlate with histological evidence of iron accumulation and ferroptotic cell death in animal models, suggesting a translational link between preclinical and clinical observations [58].

Third, the timing of reperfusion influences the extent of ferroptosis-mediated injury and correlates with clinical outcomes. Patients who achieve earlier reperfusion generally have better outcomes, potentially because earlier restoration of blood flow limits the accumulation of iron and oxidative damage in the ischemic tissue. Conversely, delayed reperfusion allows for greater iron accumulation and more extensive oxidative stress, creating conditions more conducive to ferroptosis upon blood flow restoration [52]. This temporal relationship may partially explain the time-dependent benefit of mechanical thrombectomy and the phenomenon of futile recanalization in late-presenting patients [55].

Hemorrhagic transformation, a major complication following reperfusion therapy and a strong predictor of futile recanalization, provides another link to ferroptosis [56]. The extravasation of red blood cells into brain parenchyma introduces massive amounts of hemoglobin-derived iron, dramatically expanding the labile iron pool and triggering ferroptosis in peri-hematoma regions [57]. Indeed, experimental studies have shown that ferroptosis is a major mechanism of secondary brain injury following intracerebral hemorrhage, and ferroptosis inhibitors reduce perihematomal edema and improve functional outcomes. Similarly, in patients experiencing hemorrhagic transformation after thrombectomy, ferroptosis likely contributes to ongoing neuronal damage, explaining the particularly poor prognosis associated with this complication [55].

Microvascular dysfunction following reperfusion, often referred to as the “no-reflow” phenomenon, represents another aspect of futile recanalization that may involve ferroptosis [56]. Despite successful recanalization of the proximal vessel, microvascular obstruction can prevent adequate tissue perfusion. Ferroptosis of endothelial cells and pericytes in the microcirculation may contribute to this phenomenon, as lipid peroxidation damages vascular cells and promotes microvascular thrombosis. Furthermore, ferroptosis-induced release of damage-associated molecular patterns (DAMPs) from dying cells can trigger inflammatory responses that exacerbate microvascular injury [52]. These damage-associated molecular patterns (DAMPs) engage innate immune signaling pathways via pattern-recognition receptors, particularly Toll-like receptors, thereby driving microglial activation and the recruitment of peripheral immune cells into the ischemic brain. The resulting neuroinflammatory response leads to the release of pro-inflammatory cytokines such as interleukin-1β (IL-1β), tumor necrosis factor-α (TNF-α), and interleukin-6 (IL-6), which further enhance oxidative stress and perturb iron homeostasis [59]. These cytokines can upregulate transferrin receptor expression and promote hepcidin-induced ferroportin degradation, thereby reducing iron efflux and promoting intracellular iron accumulation. In parallel, inflammatory signaling pathways compromise antioxidant defense systems, notably the GPX4–glutathione axis, which facilitates unchecked lipid peroxidation and increases cellular vulnerability to ferroptosis [60]. Consequently, inflammation and ferroptosis may form a self-perpetuating pathological cycle that aggravates neuronal and microvascular injury after reperfusion, potentially contributing to futile recanalization despite successful vessel reopening.

Patient heterogeneity significantly influences ferroptosis susceptibility and treatment responses. Age-related iron accumulation and declining antioxidant defenses make elderly patients more vulnerable to ferroptotic injury [61]. Comorbidities such as diabetes mellitus induce chronic oxidative stress and may pre-sensitize tissues to ferroptosis, while chronic kidney disease alters systemic iron homeostasis. Genetic polymorphisms in ferroptosis-related genes (GPX4, SLC7A11, iron metabolism proteins) create variable baseline susceptibilities across individuals. Differences in stroke etiology—cardioembolic versus atherosclerotic versus small vessel occlusion—may influence the extent of ferroptotic injury and response to inhibitors [62, 63]. This heterogeneity necessitates personalized approaches that account for individual patient biology, comorbidities, and stroke characteristics when developing ferroptosis-targeted interventions.

## Clinical and radiological factors associated with futile recanalization

While ferroptosis represents a key molecular mechanism underlying futile recanalization, clinical outcomes following mechanical thrombectomy are influenced by a complex interplay of patient-specific, stroke-specific, and procedural factors [55]. Understanding these factors is essential for identifying high-risk patients and developing comprehensive strategies to prevent futile recanalization. Multiple studies have investigated predictors of poor outcomes despite successful reperfusion, revealing several consistent themes across demographic, clinical, radiological, and procedural domains.

### Sex differences in ferroptosis susceptibility and stroke outcomes

Sexual dimorphism in iron metabolism and oxidative stress responses represents an important but underappreciated factor influencing ferroptosis susceptibility and stroke outcomes. Premenopausal women demonstrate lower serum ferritin levels and greater iron regulatory protein activity compared to age-matched men, potentially providing inherent protection against ferroptosis-mediated injury. Estrogen’s antioxidant properties, including upregulation of GPX4 expression and enhancement of glutathione synthesis, may explain the well-documented sex differences in stroke outcomes among younger patients [64].

However, this protection diminishes after menopause, with postmenopausal women showing increased iron accumulation and oxidative vulnerability comparable to men. Sex chromosomes independently influence ferroptosis pathways, with X-linked genes encoding several iron metabolism proteins (including G6PD and ALAS2) potentially conferring different baseline susceptibilities [65]. Importantly, most preclinical ferroptosis studies have used male animals exclusively, representing a critical gap in translational validity.

Future clinical trials of ferroptosis inhibitors should prospectively examine sex-specific effects and consider hormone status as a key biological variable. Treatment algorithms may need to account for menopausal status, endogenous hormone levels, and sex-specific biomarker thresholds to optimize therapeutic efficacy across all patient populations [66].

Advanced age consistently emerges as one of the strongest independent predictors of futile recanalization, with patients over 80 years showing significantly higher rates of poor outcomes despite successful vessel reopening [58]. The biological basis for this age-related vulnerability likely involves multiple factors, including reduced vascular compliance, decreased cerebrovascular reserve, higher prevalence of comorbidities, and age-related decline in antioxidant defenses. Importantly, aging is associated with increased brain iron accumulation and reduced GPX4 expression, potentially predisposing elderly patients to more severe ferroptosis-mediated injury following reperfusion.

Baseline stroke severity, quantified by the National Institutes of Health Stroke Scale (NIHSS) score, represents another critical determinant of outcomes after thrombectomy. Higher NIHSS scores, reflecting more extensive neurological deficits at presentation, correlate strongly with futile recanalization [57]. This relationship is intuitive, as severe baseline deficits typically indicate larger ischemic territories and more profound tissue injury. However, the molecular mechanisms linking stroke severity to ferroptosis susceptibility remain to be fully elucidated. It is plausible that more severe ischemia creates conditions more conducive to ferroptosis, including greater iron accumulation, more extensive depletion of antioxidant reserves, and more severe mitochondrial dysfunction [58].

Neuroimaging parameters, particularly those reflecting the extent of irreversibly injured tissue and the quality of collateral circulation, are powerful predictors of thrombectomy outcomes. The Alberta Stroke Program Early CT Score (ASPECTS), which quantifies early ischemic changes on non-contrast CT or DWI MRI, inversely correlates with functional outcomes—lower ASPECTS indicates more extensive early infarction and higher risk of futile recanalization [67]. Advanced perfusion imaging techniques, including CT perfusion (CTP) and MRI perfusion, provide additional prognostic information by distinguishing the ischemic core (irreversibly injured tissue) from the penumbra (at-risk but potentially salvageable tissue) [68]. Patients with large core volumes (typically defined as >70 mL) show substantially higher rates of futile recanalization, even with successful reperfusion [69].

Collateral circulation status plays a pivotal role in determining tissue fate during ischemia and outcomes following reperfusion. Good collaterals maintain some blood flow to the ischemic territory, limiting the extent of core infarction and preserving penumbral tissue that can be salvaged with timely reperfusion [70]. Conversely, poor collaterals allow rapid infarct expansion and tissue damage, reducing the opportunity for meaningful recovery despite successful thrombectomy. Collateral grading systems, such as the American Society of Interventional and Therapeutic Neuroradiology/Society of Interventional Radiology (ASITN/SIR) scale and the Miteff scale, have been developed to standardize collateral assessment and predict outcomes [71].

The time from symptom onset to reperfusion remains a fundamental determinant of outcomes, embodying the principle of “time is brain.” Each minute of delay results in the loss of approximately 1.9 million neurons, emphasizing the critical importance of rapid treatment [72]. However, the relationship between time and outcomes is not simply linear. Recent trials have demonstrated that carefully selected patients can benefit from thrombectomy even in extended time windows (up to 24 h), challenging traditional time-based paradigms and shifting focus toward tissue-based selection criteria [73]. Nevertheless, even in these extended windows, faster treatment times within the eligible period correlate with better outcomes, and delayed reperfusion increases the risk of futile recanalization [67].

Procedural factors, including the quality of recanalization achieved and the number of device passes required, influence outcomes. The modified Thrombolysis in Cerebral Infarction (mTICI) scale grades recanalization success, with mTICI 3 (complete reperfusion) associated with better outcomes compared to mTICI 2b (partial reperfusion of >50% but <100% of territory) [68]. However, even among patients achieving mTICI 2b-3, substantial heterogeneity in functional outcomes exists, underscoring the multifactorial nature of futile recanalization [69]. The number of thrombectomy passes required to achieve recanalization also matters, with first-pass complete reperfusion associated with superior outcomes compared to multiple-pass reperfusion, likely due to reduced endothelial injury and shorter procedure times [70].

Beyond traditional 90-day mRS outcomes, ferroptosis may specifically impact cognitive domains through injury to hippocampal regions that are particularly vulnerable due to high iron content and metabolic activity [74]. These regions are critical for memory consolidation and executive function. Emerging evidence links ferroptosis to post-stroke cognitive impairment and dementia risk, which profoundly affects quality of life independent of motor recovery. Post-stroke depression, affecting up to 30–40% of stroke survivors, involves oxidative stress and inflammation closely linked to ferroptosis pathways. Preventing futile recanalization through ferroptosis inhibition could reduce severe disability (mRS 4-5) that creates substantial caregiver burden and healthcare utilization [75].

Ferroptosis may limit neuroplasticity during the recovery phase, as ongoing ferroptotic injury could impair synaptic reorganization and functional compensation. Future clinical trials should incorporate comprehensive neuropsychological testing, quality-of-life assessments (such as Stroke-Specific Quality of Life scale), and caregiver burden measures alongside traditional functional outcomes. Meaningful recovery encompasses cognitive, emotional, and social dimensions beyond physical independence, aligning mechanistic insights with outcomes that truly matter to patients and families [32].

## Predictive models for futile recanalization

The development of predictive models to identify patients at high risk for futile recanalization represents a critical area of investigation, offering the potential to refine patient selection, guide treatment decisions, and stratify patients in clinical trials. These models integrate multiple variables across clinical, radiological, and laboratory domains to generate individualized risk estimates [71]. The past decade has witnessed substantial progress in this field, with the emergence of traditional statistical models (primarily nomograms) and more recently, machine learning-based approaches that can capture complex, non-linear relationships between predictor variables [76].

Several nomogram-based models have been developed and validated for predicting futile recanalization. These models typically incorporate readily available variables such as age, baseline NIHSS score, ASPECTS, glucose levels, atrial fibrillation status, time to treatment, and recanalization grade [72]. The SPAN-100 index, calculated as stroke severity (NIHSS) × age, has been widely used as a simple screening tool, with values ≥ 100 identifying patients at higher risk for poor outcomes despite successful recanalization [73]. However, while simple scoring systems offer ease of use, they may not capture the full complexity of factors influencing outcomes and generally show moderate discriminative performance with area under the curve (AUC) values of 0.65–0.75 [77].

More sophisticated nomograms have been constructed using multivariable logistic regression, incorporating additional parameters such as collateral status, white blood cell count, blood pressure variability, and comorbidities [67]. These comprehensive models show improved predictive accuracy compared to simpler scoring systems, with AUC values reaching 0.75–0.85 in derivation cohorts. However, external validation in independent cohorts often reveals reduced performance, highlighting challenges in model generalizability across different populations and practice settings [68].

Rigorous validation strategies are essential for clinical translation of predictive models. Internal validation approaches including k-fold cross-validation (typically 5- or 10-fold) and bootstrap resampling techniques (commonly 500–1,000 iterations) help assess model stability and reduce overfitting risk [53]. However, internal validation alone is insufficient for establishing clinical utility. External validation, testing models in independent cohorts from different institutions or geographic regions, represents the gold standard for assessing generalizability. Most published futile recanalization models show 10–15% decreases in AUC when tested externally, declining from 0.75 to 0.85 in derivation cohorts to 0.65–0.75 in validation cohorts [64].

This performance degradation reflects several factors: (1) differences in patient populations (geographic, demographic, and stroke severity variations); (2) heterogeneity in imaging protocols and interpretation; (3) variability in thrombectomy techniques and operator experience; and (4) differences in post-procedural care pathways. The reduced generalizability highlights critical challenges for clinical implementation. Models developed in comprehensive stroke centers with advanced imaging capabilities may not perform well in community hospitals with limited resources [77]. Prospective, multicenter validation studies are essential before widespread clinical adoption. Collaborative international registries could facilitate more robust external validation by providing large, diverse patient cohorts that better represent real-world practice heterogeneity.

The advent of machine learning has opened new avenues for predictive modeling in stroke. Machine learning algorithms, including random forests, gradient boosting machines, support vector machines, and neural networks, can automatically learn complex patterns from data without requiring explicit specification of variable relationships [78]. These approaches have shown promising results in predicting futile recanalization, with some studies reporting AUC values exceeding 0.85 [79]. Feature importance analyses from these models have identified novel predictors and confirmed the significance of established factors, providing insights into the multifaceted nature of futile recanalization.

Recent studies have begun incorporating ferroptosis-related biomarkers into predictive models, offering the potential for improved risk stratification. Serum ferritin, lipid peroxidation products, and genetic polymorphisms in ferroptosis-related genes (such as GPX4 and SLC7A11) have shown associations with outcomes after thrombectomy [79]. Machine learning models incorporating these biomarkers alongside traditional clinical and radiological variables demonstrate enhanced predictive performance, suggesting that ferroptosis markers provide complementary prognostic information. However, most of these biomarker-enriched models remain in early validation stages and require larger, prospective studies to confirm their clinical utility [80].

Despite these advances, several challenges remain in translating predictive models into routine clinical practice. First, many models lack external validation in diverse populations and healthcare settings, limiting confidence in their generalizability. Second, the optimal threshold for defining “futile recanalization” varies across studies, with some using mRS 3–6 and others using mRS 4–6, complicating comparisons and meta-analyses [81]. Third, most models focus on 90-day functional outcomes, but earlier prediction of outcomes could inform acute management decisions, such as intensity of monitoring or timing of rehabilitation.

Future directions in predictive modeling include the integration of multimodal data, including advanced neuroimaging features (e.g., texture analysis, deep learning-based image interpretation), multi-omic biomarkers (genomics, proteomics, metabolomics), and real-time procedural parameters [82]. Dynamic prediction models that update risk estimates during the course of treatment, incorporating procedural outcomes and early post-procedural data, may offer more nuanced guidance for individualized care. Additionally, the development of explainable AI models that provide transparent reasoning for predictions will be essential for clinical acceptance and implementation [79].

## Therapeutic interventions targeting ferroptosis and futile recanalization

The recognition of ferroptosis as a critical mechanism in cerebral ischemia-reperfusion injury and futile recanalization has spurred intense interest in developing therapeutic strategies to inhibit this cell death pathway. Potential interventions span multiple levels, from modulating iron metabolism and enhancing antioxidant defenses to directly inhibiting lipid peroxidation and promoting membrane repair. While most anti-ferroptotic therapies remain in preclinical stages, some have shown sufficient promise to warrant clinical investigation [82].

### Iron chelation therapy

Iron chelation represents one of the most direct approaches to preventing ferroptosis by reducing the availability of catalytically active iron. Deferoxamine (DFO), an FDA-approved iron chelator used for treating iron overload conditions, has shown neuroprotective effects in experimental stroke models. DFO chelates free iron, preventing its participation in Fenton chemistry and subsequent lipid peroxidation. In rodent MCAO models, DFO administration reduced infarct volume, improved functional outcomes, and decreased markers of ferroptosis when given during or shortly after reperfusion [80].

Small clinical studies have explored DFO in intracerebral hemorrhage patients, where iron-mediated injury is prominent, showing safety and potential efficacy signals. However, DFO has limitations, including poor blood-brain barrier penetration, short half-life requiring continuous infusion, and potential side effects with high-dose or prolonged use. Newer iron chelators with improved pharmacokinetic properties, such as deferiprone and deferasirox, have shown promise in preclinical studies and may offer advantages for clinical translation [83].

Alternative strategies to modulate iron metabolism include targeting specific components of the iron regulatory pathway. Inhibition of ferritinophagy, either through NCOA4 knockdown or lysosomal inhibition, has demonstrated neuroprotective effects by preventing the liberation of iron from ferritin stores [80]. Upregulation of ferroportin expression or prevention of hepcidin-mediated ferroportin degradation could enhance cellular iron export, reducing intracellular iron accumulation. These more targeted approaches may offer advantages over broad iron chelation by preserving essential iron-dependent processes while specifically blocking pathological iron accumulation [82].

Several ferroptosis-targeting compounds have advanced beyond preclinical stages, providing important translational insights. Deferoxamine has been tested in the i-DEF trial (NCT00777140), a completed phase II study in intracerebral hemorrhage patients that demonstrated safety but mixed efficacy signals. Ongoing investigations are exploring deferoxamine in ischemic stroke populations, though optimal dosing and timing remain uncertain [53]. N-acetylcysteine (NAC), which replenishes glutathione stores, has been evaluated in recent phase II trials in acute ischemic stroke, demonstrating safety with high-dose intravenous administration (up to 70 mg/kg loading dose followed by 20 mg/kg/hour infusion), though definitive efficacy trials are still needed [63].

Regarding selenium supplementation, observational studies show associations between selenium status and stroke outcomes, but large-scale randomized controlled trials specifically testing selenium in acute stroke are lacking. Novel ferroptosis inhibitors are entering clinical development, including liproxstatin-1 analogs being optimized for improved pharmacokinetics and blood-brain barrier penetration [84]. These compounds show promising preclinical neuroprotective effects, but extensive safety testing is required before human trials. The translation requires addressing challenges including optimal timing relative to reperfusion, identification of biomarkers for patient selection, and development of combination strategies that target multiple ferroptosis pathways simultaneously [54].

### Lipophilic antioxidants and ferroptosis inhibitors

Lipophilic radical-trapping antioxidants (RTAs) directly inhibit ferroptosis by intercepting lipid peroxyl radicals and breaking the chain reaction of lipid peroxidation. Ferrostatin-1 (Fer-1), the prototypical ferroptosis inhibitor discovered during the initial characterization of ferroptosis, has shown robust neuroprotective effects in numerous experimental stroke models. Fer-1 functions by directly reducing lipid peroxyl radicals in membranes, preventing the propagation of lipid peroxidation. Importantly, Fer-1 provides protection even when administered hours after ischemia onset, highlighting a therapeutic window that extends well beyond the acute reperfusion phase [80].

Liproxstatin-1 (Lip-1), a more potent analog of Fer-1 with improved pharmacokinetic properties, demonstrates enhanced efficacy in preventing ferroptosis-mediated neuronal death. Lip-1 exhibits better blood-brain barrier penetration and longer half-life compared to Fer-1, making it more suitable for clinical development. In MCAO models with reperfusion, Lip-1 treatment significantly reduced infarct volume, preserved penumbral tissue, and improved neurobehavioral outcomes [85].

Vitamin E (α-tocopherol), a lipid-soluble antioxidant that has been used safely in humans for decades, functions as a chain-breaking antioxidant by donating hydrogen atoms to lipid peroxyl radicals. While vitamin E has shown mixed results in clinical stroke trials when used alone, its combination with other antioxidants or ferroptosis inhibitors may offer synergistic neuroprotection. Tocotrienols, unsaturated forms of vitamin E with enhanced antioxidant activity, have demonstrated superior neuroprotective effects in experimental models and warrant further investigation [86].

CoQ10 and its reduced form, ubiquinol, serve dual roles as mitochondrial electron transport chain components and lipophilic antioxidants capable of preventing lipid peroxidation [73]. Given the newly discovered FSP1-CoQ10 and DHODH-CoQ10 anti-ferroptotic pathways, therapeutic strategies to enhance CoQ10 availability may offer particular promise. CoQ10 supplementation has shown safety in numerous clinical studies, though its efficacy in acute stroke requires rigorous evaluation [85].

### Enhancing GPX4 activity and glutathione availability

Strategies to enhance GPX4 expression and activity represent logical approaches to preventing ferroptosis, given GPX4’s central role as the primary defense against lipid peroxidation. Selenium supplementation can enhance GPX4 activity by ensuring adequate incorporation of selenocysteine into the enzyme’s catalytic site. Clinical studies have shown associations between selenium status and stroke outcomes, with selenium deficiency linked to increased stroke severity and poorer recovery. However, optimal dosing and timing of selenium supplementation in acute stroke remain to be established, and excessive selenium can be toxic [86].

Increasing GSH availability represents another avenue for enhancing GPX4-dependent ferroptosis resistance. N-acetylcysteine (NAC), a precursor to cysteine and GSH, has been widely used as an antioxidant and mucolytic agent with an excellent safety profile [87]. NAC can replenish cellular GSH stores, supporting GPX4 activity and enhancing overall antioxidant capacity. Clinical trials of NAC in acute stroke have shown mixed results, potentially due to suboptimal dosing or timing, but the compound’s safety and theoretical rationale support continued investigation [86].

Modulation of system xc^-^ activity presents a more nuanced challenge. While inhibition of system xc^-^ induces ferroptosis in experimental settings, enhancing its activity may protect against ferroptosis by promoting cystine uptake and GSH synthesis. However, system xc^-^ upregulation could potentially exacerbate glutamate excitotoxicity, a competing mechanism of ischemic injury. This complex interplay highlights the need for careful optimization of therapeutic strategies targeting system xc^-^ in stroke [87].

Activation of the Nrf2 pathway, which transcriptionally upregulates multiple antioxidant genes including SLC7A11, GPX4, and glutamate-cysteine ligase, offers a coordinated approach to enhancing cellular anti-ferroptotic defenses [86]. Several Nrf2 activators, including dimethyl fumarate (DMF) and sulforaphane, have shown neuroprotective effects in experimental stroke models. DMF is already FDA-approved for multiple sclerosis treatment, potentially facilitating its repurposing for stroke neuroprotection [87].

## Future perspectives and clinical translation

The integration of ferroptosis biology into our understanding of ischemic stroke pathophysiology and futile recanalization represents a paradigm shift with significant implications for future research and clinical practice [86]. Moving forward, several key priorities emerge that could accelerate the translation of ferroptosis-targeted therapies from bench to bedside and ultimately improve outcomes for stroke patients undergoing mechanical thrombectomy [88].

### Implementation challenges and health economics considerations

The proposed integration of ferroptosis biomarkers, advanced multimodal neuroimaging, and machine learning-based predictive models requires substantial infrastructure investment that may be prohibitive in many healthcare settings. Low-resource settings might initially focus on simple, readily available markers such as serum ferritin and basic iron panels, while reserving sophisticated multimodal approaches for well-resourced comprehensive stroke centers. Preventing futile recanalization could substantially reduce long-term healthcare costs associated with severe disability, potentially offsetting upfront diagnostic and therapeutic expenses. However, formal cost-effectiveness analyses are currently lacking and represent a critical research priority [53].

Global health equity concerns are paramount, as low- and middle-income countries bearing the highest stroke burden often lack access even to basic thrombectomy services, let alone advanced ferroptosis-targeted interventions. International collaborations should focus on developing affordable, point-of-care ferroptosis biomarker assays and simplified risk stratification tools that can be implemented in resource-limited settings [75]. This approach grounds our translational framework in practical healthcare delivery realities and acknowledges the economic constraints that will ultimately determine clinical adoption of ferroptosis-targeted strategies.

### Translational challenges and the validity gap in ferroptosis research

Most preclinical ferroptosis studies employ young (8–12 week old), genetically uniform rodents subjected to standardized temporary MCAO, which poorly represents the elderly, heterogeneous stroke population with multiple comorbidities [89]. Specific translational concerns include: (1) aging-related changes, noting that elderly patients demonstrate baseline iron accumulation, reduced GPX4 expression, impaired autophagy, and chronic oxidative stress that fundamentally alter ferroptosis susceptibility; (2) comorbidity effects, with diabetes causing chronic hyperglycemia and oxidative stress that may pre-sensitize neurons, while chronic kidney disease alters systemic iron handling; (3) pre-existing white matter disease and cerebral small vessel disease that modify tissue vulnerability; (4) polypharmacy effects, as commonly used medications (statins, antihypertensives, anticoagulants) may interact with ferroptosis pathways unpredictably [74].

Development of aged animal models with relevant comorbidities (diabetic, hypertensive strains) and incorporation of chronic treatment paradigms are essential for more clinically relevant translational research. Early-phase clinical trials should specifically recruit patients representative of real-world stroke populations rather than highly selected cohorts, and translational studies should systematically examine how aging and comorbidities modify responses to ferroptosis inhibition [62]. This critical self-reflection acknowledges fundamental limitations in the current evidence base and provides a roadmap for more clinically relevant research.

First, the development and validation of clinically applicable ferroptosis biomarkers is essential for identifying patients at highest risk and monitoring treatment responses. While experimental studies have identified numerous ferroptosis markers in brain tissue, translating these findings to accessible biomarkers in blood or cerebrospinal fluid remains challenging. Potential candidates include circulating levels of lipid peroxidation products (MDA, 4-HNE), iron metabolism markers (ferritin, transferrin saturation, hepcidin), and emerging omics-based signatures (ferroptosis-related gene expression panels, metabolomic profiles) [90]. Prospective studies correlating these biomarkers with clinical outcomes in well-characterized patient cohorts are needed to establish their prognostic value.

Second, advanced neuroimaging techniques capable of detecting ferroptosis *in vivo* would enable real-time assessment of ferroptotic injury and guide therapeutic interventions. Quantitative susceptibility mapping (QSM) shows promise for detecting iron accumulation in the brain and has already been applied in stroke patients [91]. The development of PET or SPECT tracers specific for ferroptosis markers, such as lipid peroxidation products or ferroptosis-related proteins, could provide dynamic imaging of ferroptotic cell death. Integration of these imaging biomarkers into predictive models may substantially improve risk stratification and treatment selection [92].

Third, rigorous clinical trials of ferroptosis inhibitors in acute ischemic stroke are urgently needed. Several candidates with favorable safety profiles in other indications (e.g., DFO, NAC, vitamin E, selenium, CoQ10) could be rapidly moved into phase II trials. Novel compounds with superior pharmacokinetic properties and greater specificity for ferroptosis pathways are in preclinical development and should be prioritized for clinical translation [93]. Trial design considerations include optimal timing of intervention (during thrombectomy, immediately post-procedure, or in the subsequent hours to days), dosing regimens, and selection of appropriate patient populations most likely to benefit [94].

Fourth, combination therapeutic strategies that target multiple aspects of reperfusion injury, including ferroptosis, excitotoxicity, inflammation, and oxidative stress, may offer synergistic neuroprotection [94]. Historical neuroprotection trials have largely focused on single mechanisms, potentially explaining their failure to demonstrate clinical benefit. A precision medicine approach that matches specific neuroprotective strategies to individual patient biology and injury patterns, informed by biomarkers and predictive models, may finally succeed where previous one-size-fits-all approaches have failed [95].

Finally, the investigation of ferroptosis extends beyond the acute reperfusion period, with potential relevance to stroke recovery and long-term neuroplasticity. Chronic neuroinflammation and oxidative stress persist for weeks to months after stroke, potentially contributing to ongoing neuronal loss and limiting recovery. Whether ferroptosis continues to play a role in this chronic phase and whether sustained ferroptosis inhibition could enhance recovery remains to be determined [90]. Additionally, understanding how ferroptosis and its inhibition influence neuroplasticity, neurogenesis, and functional reorganization could inform rehabilitation strategies and optimize long-term outcomes [91].

## Discussion

### Overall interpretation of the evidence

Futile recanalization following mechanical thrombectomy for acute ischemic stroke represents a critical challenge that limits the effectiveness of an otherwise revolutionary treatment. The emergence of ferroptosis as a key mechanism underlying reperfusion injury and poor outcomes provides new insights into this phenomenon and offers novel therapeutic targets. Ferroptosis, characterized by iron-dependent lipid peroxidation, is driven by disruption of iron homeostasis, accumulation of oxidized phospholipids, and failure of antioxidant defense systems, particularly the GPX4-GSH axis. Multiple lines of evidence from experimental and clinical studies support the involvement of ferroptosis in ischemic stroke, especially in the context of reperfusion injury following mechanical thrombectomy.

### Clinical implications

A comprehensive understanding of futile recanalization requires integration of ferroptosis mechanisms with established clinical, radiological, and procedural factors. Patient characteristics (age, comorbidities), stroke features (severity, infarct size, collateral status), timing of treatment, and quality of reperfusion all interact to determine outcomes. Predictive models incorporating these multiple dimensions, potentially enhanced by ferroptosis biomarkers, offer promise for improved risk stratification and personalized treatment strategies. Therapeutic interventions targeting ferroptosis, including iron chelation, lipophilic antioxidants, and strategies to enhance GPX4 activity, have shown efficacy in preclinical models and warrant clinical investigation.

### Future directions

Moving forward, the field requires continued collaboration between basic scientists, clinical researchers, and industry partners to translate ferroptosis biology into actionable clinical applications. The development of validated biomarkers, advanced imaging techniques, and targeted therapies specifically designed for the unique pathophysiology of ischemic stroke with reperfusion will be essential. With the growing recognition that successful recanalization is necessary but not sufficient for good outcomes, addressing the molecular mechanisms of reperfusion injury, particularly ferroptosis, may finally bridge the gap between technical success and meaningful functional recovery.

### Conclusion

By combining optimal revascularization techniques with targeted strategies aimed at preventing or attenuating ferroptosis-mediated neuronal injury and its downstream pathological consequences, it may be possible to reduce the incidence of futile recanalization and maximize the benefit of mechanical thrombectomy for stroke patients.
